# Engineered Hyperactive Integrase for Concerted HIV-1 DNA Integration

**DOI:** 10.1371/journal.pone.0105078

**Published:** 2014-08-13

**Authors:** Min Li, Kellie A. Jurado, Shiqiang Lin, Alan Engelman, Robert Craigie

**Affiliations:** 1 Laboratory of Molecular Biology, National Institute of Diabetes and Digestive and Kidney Diseases, National Institutes of Health, Bethesda, Maryland, United States of America; 2 Department of Cancer Immunology and AIDS, Dana-Farber Cancer Institute and Department of Medicine, Harvard Medical School, Boston, Massachusetts, United States of America; Centro de Biología Molecular Severo Ochoa (CSIC-UAM), Spain

## Abstract

The DNA cutting and joining reactions of HIV-1 integration are catalyzed by integrase (IN), a viral protein that functions as a tetramer bridging the two viral DNA ends (intasome). Two major obstacles for biochemical and structural studies of HIV-1 intasomes are 1) the low efficiency of assembly with oligonucleotide DNA substrates, and 2) the non-specific aggregation of both intasomes and free IN in the reaction mixture. By fusing IN with a small non-specific DNA binding protein, *Sulfolobus solfataricus* chromosomal protein Sso7d (PDB: 1BNZ), we have engineered a highly soluble and hyperactive IN. Unlike wild-type IN, it efficiently catalyzes intasome assembly and concerted integration with oligonucleotide DNA substrates. The fusion IN protein also functions to integrate viral reverse transcripts during HIV-infection. The hyperactive HIV-1 IN may assist in facilitating future biochemical and structural studies of HIV-1 intasomes. Understanding the mechanistic basis of the Sso7d-IN fusion protein could provide insight into the factors that have hindered biophysical studies of wild-type HIV-1 IN and intasomes.

## Introduction

Integration of retroviral DNA into the host chromosomal DNA is an essential step in the retroviral replication cycle (reviewed in [1]). The newly synthesized viral DNA is initially blunt ended, yet prior to integration into cellular DNA it must be processed by the removal of two nucleotides from each 3′ end. The 3′ end processing reaction exposes the 3′ hydroxyl groups that are used in the subsequent attack of phosphodiester bonds at the site of integration into host chromosomal DNA within the nucleus during the DNA strand transfer reaction. In the case of HIV, the sites of insertion on the two target DNA strands are separated by 5 bp, resulting in a 5 bp duplication of target DNA sequence flanking the integrated provirus upon repair of the integration intermediate.

Under most reaction conditions HIV-1 integrase (IN) predominantly catalyzes a half-site reaction in which only a single viral DNA end is joined to one strand of target DNA, rather than the two-ended reaction that is required for productive integration. In contrast, preintegration complexes (PICs) isolated from infected cells exclusively carry out two-end integration *in vitro*. Improved reaction conditions support concerted integration [2]–[4], but the efficiency is low and both the substrates and products aggregate. Concerted integration proceeds through a series of stable nucleoprotein complexes, or intasomes [5]. First, a tetramer of IN bridges the pair of newly reverse-transcribed viral DNA ends to form the Stable Synaptic Complex (SSC). Processing of the viral DNA ends converts the SSC to the cleaved intasome (CI) [6] or cleaved donor complex (CDC) [7]. Next, subsequent to nuclear import, the CI captures a target DNA and covalently joins viral to target DNA. The product DNA remains associated with the IN tetramer in a stable complex called the Strand Transfer Complex (STC). IN inhibitors such as Raltegravir and Dolutegravir recognize intasomes rather than free IN protein [8], so high resolution structures of intasomes are needed to understand the atomic details of the mechanism of inhibition and evolution of resistance. To date there are no high-resolution structures of HIV intasomes, although structures of the closely related Prototype Foamy Virus (PFV) intasomes have been determined [6], [9], [10]. The PFV structures serve as an excellent guide to model the active site of HIV intasomes [11], [12], but sequence divergence makes modeling less reliable outside the immediate vicinity of the active site.

The major obstacles to high-resolution structural studies of HIV intasomes are: 1) the low efficiency of assembly with oligonucleotide DNA substrates, and 2) non-specific aggregation of both intasomes and free IN in the reaction mixture. In contrast, PFV intasomes are soluble and monodisperse at high concentration and are efficiently assembled with oligonucleotide DNA [10], [13]. A striking feature of PFV IN is the presence of an extra domain, the N-terminal extension domain (NED), which interacts with viral DNA in the PFV intasome structures. We therefore tested whether fusing non-specific DNA binding domains to the N-terminus of HIV-1 IN would confer some of the favorable properties of PFV IN. One of the domains we tested, *Sulfolobus solfataricus* chromosomal protein Sso7d (PDB: 1BNZ), resulted in a hyperactive IN protein. Unlike wild-type IN, it efficiently catalyzes intasome assembly and concerted integration with oligonucleotide DNA substrates *in vitro*. The intasomes are also much more soluble than those assembled with the wild-type protein. The hyperactive HIV IN will facilitate future biochemical and structural studies of HIV intasomes.

## Materials and Methods

### DNA substrates and recombinant DNA construction

The oligonucleotide viral DNA substrates used in this work contained 10 bp of a GC rich motif (AGCGTGGGCG) at the 5′ end, followed by U5 DNA sequence from the long terminal repeat (LTR) terminus. Double strand pre-processed DNA substrates were: U5-19, AGCGTGGGCGTCTCTAGCA; U5-21, AGCGTGGGCGAATCTCTAGCA; U5-23, AGCGTGGGCGAAAATCTCTAGCA; U5-25, AGCGTGGGCGGGAAAATCTCTAGCA; U5-27, AGCGTGGGCGGTGGAAAATCTCTAGCA; U5-29, AGCGTGGGCGGTGTGGAAAATCTCTAGCA; U5-33, AGCGTGGGCGGTCAGTGTGGAAAATCTCTAGCA; U5-37, AGCGTGGGCGTTTAGTCAGTGTGGAAAATCTCTAGCA; U5-41, AGCGTGGGCGCCCTTTTAGTCAGTGTGGAAAATCTCTAGCA.

U5-25 was used for the experiments, unless otherwise noted. Fluorescent DNA substrates were prepared by attaching 6-FAM fluorophor at the 5′ ends of the above oligonucleotides. Oligonucleotides were purchased from Integrated DNA Technologies (Coralville, Iowa). An N-terminal His-tagged Sso7d fusion to the HIV-1 IN sequence (Sso7d-IN) was synthesised by GenScript (Piscataway, NJ) in pET-28a. Various lengths of glycine linker were introduced between the Sso7d and IN domains. Sso7d-IN with an 11 glycine linker (referred as to Gly-11) was used for this work unless otherwise noted. A DNA binding deficient mutant, Sso7d_mut,_ harbored two point mutations, W24A and R43E. Vpr fusion (Vpr-IN) constructs were prepared by insertion of Sso7d-IN or Sso7d_mut_-IN DNA into pLR2P-Vpr vector DNA between the BamHI and XhoI sites; the constructs maintained the upstream PR cleavage site IRKVL/FLDGI.

### Protein expression and purification

His-tagged wild-type IN and Sso7d-IN were expressed and purified essentially as described [5] with minor modifications. Briefly, the IN was expressed in *E. coli* BL21(DE3) and the cells were lysed in buffer containing 20 mM Hepes pH 7.5, 10% glycerol, 2 mM 2-mercaptoethanol, 20 mM imidazole and 1 M NaCl. The protein was purified by nickel-affinity chromatography and the His-tag was removed with thrombin. Aggregated protein was removed by gel filtration on a Hiload 26/60 Superdex-200 column (GE Healthcare) equilibrated with 20 mM Hepes pH 7.5, 10% glycerol, 5 mM DTT, 1 mM EDTA and 1 M NaCl. The protein was concentrated using an Amicon centrifugal contentrator (EMD Millipore) as necessary, flash-frozen in liquid nirogen and stored at −80°C.

### Integration assay and intasome assembly

IN (1 µM, unless otherwise noted) and 0.5 µM viral DNA substrate were preincubated on ice in 20 mM HEPES pH 7.5, 25% glycerol, 10 mM DTT, 5 mM MgCl_2_, 4 µM ZnCl_2_, and 100 mM NaCl in a 20 µl reaction volume. 300 ng of target plasmid DNA pGEM-9zf was then added and the reaction was initiated by transfer to 37°C and incubation for 1 hr. For integration product analysis, the reactions were stopped by addition of SDS and EDTA to 0.2% and 10 mM, respectively, together with 5 µg of proteinase K. Incubation was continued at 37°C for a further 1 hr. The DNA was then recovered by ethanol precipatation and subjected to electrophoresis in a 1.5% agarose gel in 1x TBE buffer. DNA was visualized either by ethidium bromide staining or by fluorescence using a Typhoon 8600 fluorescence scanner (GE Healthcare). Intasome assembly was carried out in the same way except that no target DNA was added and CaCl_2_ was substituted for MgCl_2_. For electrophoretic mobility shift assays of intasomes (EMSA), the reaction was stopped after 1 hr incubation at 37°C by chilling on ice and addition of 10 µg/ml heparin. A 2.5 µl aliquot was subjected to electrophoresis on a 3.0% low melting 1x TBE agarose gel (SeaKem LE agarose) containing 10 µg/ml heparin. Integration products were sequenced as described [14]. Briefly, linear DNA corresponding to concerted integration products was isolated from an agarose gel and ligated to the Tn5 aminoglycoside-3′-*O*-phosphotransferase (kanamycin resistance cassette) gene. The DNA was then transformed into *E. coli*, and the plasmids were recovered from kanamycin resistant colonies. Plasmids with the expected correct size were sequenced and analyzed.

### Size-exclusion chromatography of intasomes

500 mM NaCl was added to scaled-up intasome assembly reaction mixtures (100 µl). After incubation at RT for 15 min, the mixture was centrifuged at 15,000 g for 15 min and the supernatant was concentrated to 50 µl using a micro concentrator (Satorius Stedim Biotech) and loaded onto a Superdex 200 PC 3.2/30 gel filtration column (GE Healthcare) equlibrated with 20 mM HEPES pH 7.5, 20% glycerol, 5 mM DTT and 500 mM NaCl. The flow rate was 40 µl/min and the fraction size was 50 µl. Fractions were assayed for integration activity. Briefly, 20 µl of each fraction was added to a 80 µl reaction mixture containing 20 mM HEPES pH 7.5, 25% glycerol, 10 mM DTT, 5 mM MgCl_2_, 4 µM ZnCl_2_, and 300 ng of pGEM-9zf. The NaCl concentration was adjusted to 100 mM. Integration products were analyzed as described above.

### Infectivity assay for Sso7d-IN function

The infectivity assay is based on the ability of IN expressed as a Vpr fusion protein to trans-complement virus lacking a functional integrase in a single round of infection. Plasmids pNLX.Luc.R- [15], pN/N.Luc(R-) [15], and pNLX.Luc.R-ΔIN [16] expressed single-round HIV-1_NL4-3_ carrying wild-type IN, D64N/D116N IN active site mutations, or a stop codon between the RT and IN boundary, respectively. Plasmid pRL2PVpr-IN expressed Vpr fused to the IN protein from HIV-1_SG3_ [17]. The D64A missense mutation was introduced into the IN coding region of pRL2PVpr-IN using PCR-directed mutagenesis. pRL2PVpr-IN was modified by replacing the coding sequence of SG3 IN with that of NL4-3 IN. The coding sequence for Sso7d was inserted into each plasmid so as the fuse Sso7d to the N-terminus of IN with a 11 aa glycine linker. Plasmid pCG-VSV-G [15] was used to express the vesicular stomatitis virus G (VSV-G) glycoprotein.

HEK293T cells were grown in Dulbecco’s modified Eagle medium supplemented to contain 10% (v/v) fetal bovine serum, 100 IU/mL penicillin, and 100 µg/mL streptomycin. Pseudovirions harboring transcomplemented IN were constructed by co-transfecting HEK293T cells with pRL2PVpr-IN expression plasmids as described [16]. Cell-free supernatants were measured for p24 content utilizing a commercial p24 ELISA kit (Advanced Biosciences Laboratories), and SupT1 T cells were infected with p24-normalized levels of virus as described [16]. Raltegravir (RAL; 10 µM), which was obtained from the National Institutes of Health AIDS Research and Reference Reagent Program, was added to target cells at the time of infection.

Supernatants for immunoblotting were pelleted via ultracentrifugation at 4°C for 2 h in a Beckman SW41 rotor at 32,000 rpm. Pelleted virions were lysed in 40 µl SDS/PAGE sample buffer [0.3125 M Tris-HCl pH 6.8, 2% SDS, 10% (wt/vol) glycerol, 5% (wt/vol) 2-mercaptoethanol, 0.001% bromophenol blue, 0.1 M dithiothreitol], boiled for 5 min, and p24-normalized levels of viral lysates were fractionated by SDS/PAGE. IN and p24 were detected using mouse antibodies 8E5 [18] and anti-HIV-1 p24 (Abcam) at 1∶5,000 and 1∶1,000 dilution, respectively. Horseradish peroxidase-conjugated secondary antibodies (Dako) were used at 1∶10,000.

## Results

### Construction of a hyperactive IN mutant

**Figure 1:**
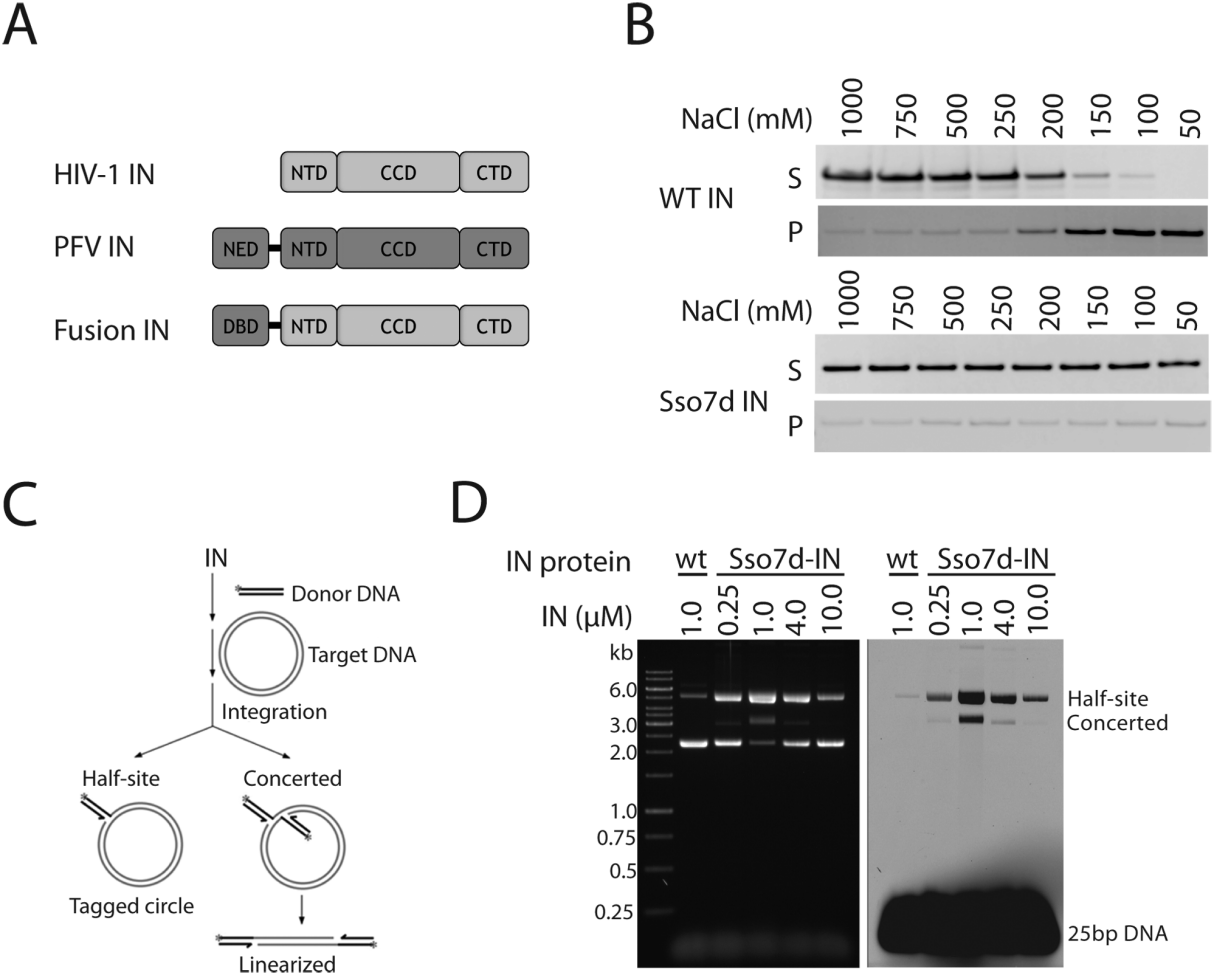
Sso7d-IN is a hyperactive IN. A, Schematic of the IN fusion proteins. NED, N-terminal extension domain NTD, N-terminal domain; CCD, catalytic core domain; CTD, C-terminal domain. B, Comparison of the solubilities of wild-type HIV-1 IN and Sso7d-IN. Proteins were incubated at the indicated NaCl concentrations in 20 mM HEPES pH 7.5, 10% glycerol, 5 mM DTT and 1 mM EDTA, centrifuged and the supernatants and pellets were analyzed by SDS PAGE. C, Schematic of the *in vitro* integration reaction with a double stranded oligonucelotide mimicking viral LTR-U5 and a circular target DNA. D, Strand transfer reaction carried with either wild-type HIV-1 IN or Sso7d-IN with an 11 amino acid linker and a fluorescently labeled viral DNA substrate (U5-25) in 20 mM HEPES pH 7.5, 10 mM DTT, 5 mM MgCl_2_, 4 µM ZnCl_2_, 100 mM NaCl, 300 ng pGEM-9zf and 0.5 µM viral DNA substrate. The position of concerted and half-site integration products is indicated. The same gel was visualized by either ethidium bromide staining (left panel) or a Typhoon 8600 fluorescence scanner (right panel).

PFV IN has several advantages over HIV IN that make it more amenable to structural studies. The protein is soluble in solution and remains so when complexed viral DNA ends in the SSC [10], [13]. Furthermore, it assembles SSCs and is highly active with short oligonucleotide viral DNA substrates. One major difference between HIV IN and PFV IN is the presence of the extra NED that binds DNA. Since other attempts to improve the properties of HIV IN by mutagenesis have been largely unsuccessful, we decided to take a long-shot approach of fusing other non-specific DNA binding domains to the N-terminus of HIV IN to make it more similar to the PFV enzyme (Figure 1A). We screened more than 30 DNA binding domains, including the PFV NED, zinc finger domains, *pur* repressor DNA binding domains, poxvirus type IB topoisomerase DNA binding domains, and Sso7d (*S. solfataricus* chromosomal protein). As expected, most of fusion proteins exhibited worse behavior and lower activity than the wild-type IN. In contrast, we found that HIV IN fused with Ssod7 behaved much better than wild-type IN and was predominantly monomeric under conditions where wild-type is extensively aggregated (data not shown). Sso7d is a small (∼7,000 KDa), chromosomal protein from the hyperthermophilic archaebacteria *S. solfataricus*. It binds to DNA non-specifically and the structure in complex with DNA has been solved [19]. Fusion of this domain to other proteins has previously been shown to confer interesting properties, including increasing the processivity of DNA polymerases [20].

### Sso7d - HIV IN is competent for highly efficient concerted integration in vitro

Wild-type IN has very low solubility in physiological buffer conditions (150 mM NaCl). In order to keep it soluble, it is usually stored in high salt (such as 1 M NaCl). We first compared the solubility of Sso7d-IN protein with that of wild-type. The same amount of each protein (3 µM) was incubated in 20 mM HEPES pH 7.5, 10% glycerol, 5 mM DTT and 1 mM EDTA in the presence of NaCl ranging from 50 mM to 1 M at room temperature for 30 min. After centrifugation at 15,000 g for 15 min, the soluble protein in the supernatant and the insoluble protein in the pellet were analyzed by SDS-PAGE. The results are shown in Figure 1B. Sso7d-IN exhibited much better solubility than wild-type IN, remaining soluble even at 150 mM and 100 mM NaCl conditions. We next analyzed the ability of Sso7d-IN to catalyze concerted DNA integration with short (25 bp) oligonucleotide viral DNA substrates containing a 6-FAM fluorophore and a circular target DNA (Figure 1C) in a buffer containing 20 mM HEPES pH 7.5, 10 mM DTT, 5 mM MgCl_2_, 4 µM ZnCl_2_, and 100 mM NaCl, in the absence of glycerol, DMSO and PEG, which are required for integration with wild-type HIV IN (Figure 1D). Concerted integration was readily detected and activity was maximal at a protein to viral DNA ratio of about 2∶1. The panel on the left is stained with ethidium bromide and in the right panel the 6-FAM fluorophore is visualized. Interestingly, mutations in Sso7d that impair DNA binding did not reduce the hyperactive phenotype (Figure S1).

### Optimization of the Sso7d-IN construct and reaction condition

**Figure 2:**
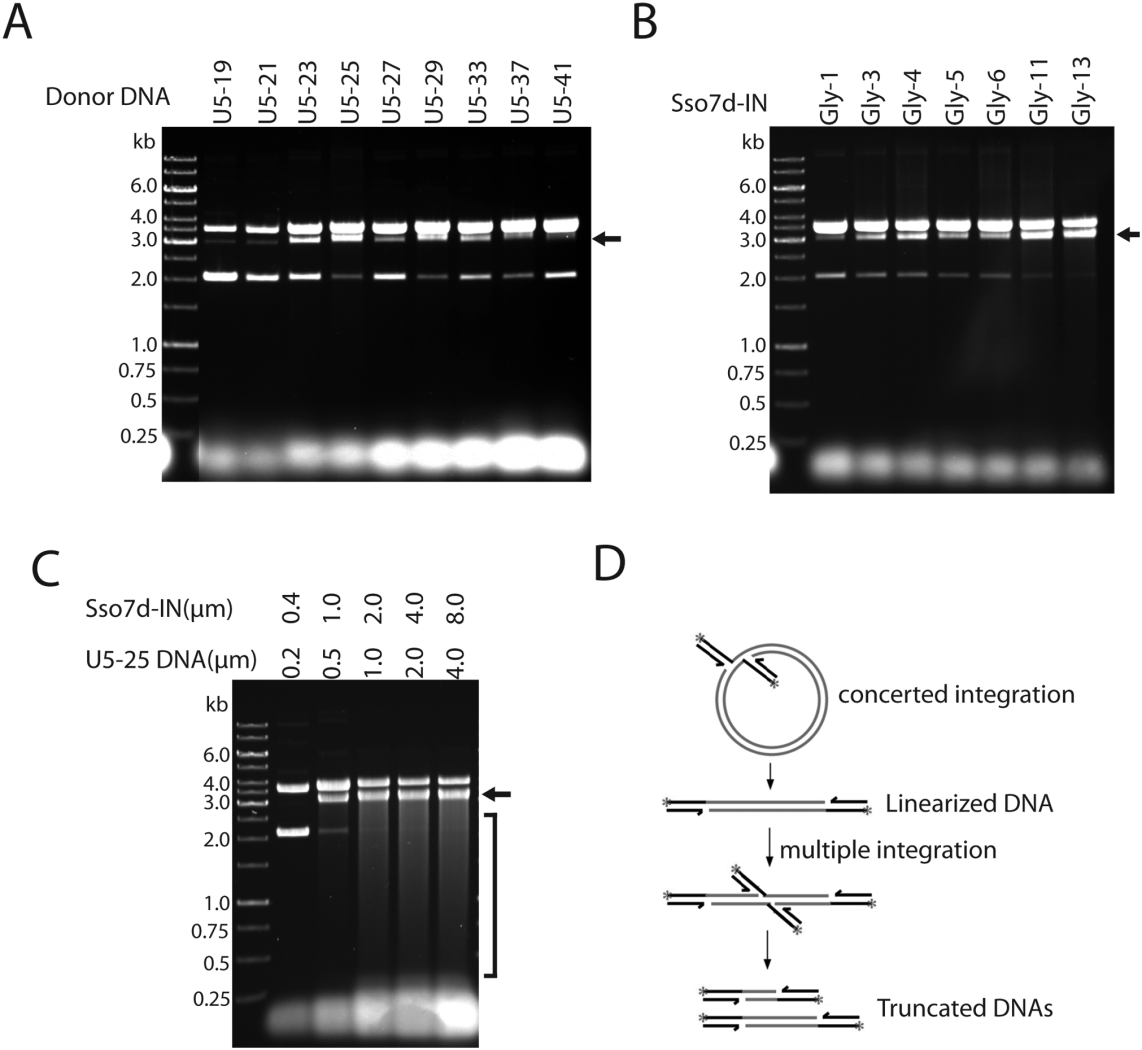
Optimization of reaction conditions with Sso7d-IN and oligonucleotide DNA substrates. Concerted integration bands are indicated with arrows. A, Effect of donor DNA length. The reactions were carried with 1 µM Sso7d-IN (Gly-11) and 0.5 µM viral DNA substrate containing a “GC rich” motif in 20 mM HEPES pH 7.5, 10 mM DTT, 5 mM MgCl_2_, 4 µM ZnCl_2_, 100 mM NaCl, and 300 ng pGEM-9zf. B, Reactions were carried with 1 µM Sso7d-IN differing in the length of the glycine linker. C, Concerted integration under optimized conditions. The ratio of Sso7d-IN (Gly-11) to donor DNA (U5-25) was kept constant at 2∶1. Sso7d-IN concentrations are 0.4 µM (lane 1), 1.0 µM (lane 2), 2.0 µM (lane 3), 4.0 µM (lane 4) and 8.0 µM (lane 5). 25% glycerol was included in the reaction buffer. The DNA smear (S) below the linear concerted integration product results from multiple integrations into the same target DNA (depicted in D).

The length of LTR-U5 DNA sequence was varied to maximize the reaction efficiency (Figure 2A). Concerted integration was minimal for DNA substrates shorter than 21 bp. DNA longer than 37 bp was less efficient, perhaps due to the competition for the fixed protein concentration in the reaction mixture. During reaction optimization of Sso7d-IN, we noticed that substrates containing the GC rich motif AGCGTGGGCG at the 5′ end of the non-joining strand had slightly greater activity (Figure S1), but this phenomenon was not further explored. The length of the linker between Sso7d and IN was also optimized (Figure 2B). Fusion proteins with a glycine linker as short as 1 amino acid (Gly-1) were capable of catalyzing concerted integration, but the reaction was somewhat more efficient with longer linkers, likely the result of greater flexibility between the domains, increasing modestly from Gly-3 to Gly-11. Unless otherwise stated, Sso7d-IN with an 11-glycine linker was used for the rest of the work. We also found the strand transfer reaction was favored in 20–25% glycerol, whereas PEG and DMSO, which stimulate the reaction with wild-type IN, reduced the reaction efficiency with Sso7d-IN (data not shown). Figure 2C shows concerted integration carried out by the Sso7d-IN with an 11 amino acid linker and U5-25 in the presence of 25% glycerol. The reaction is so efficient that the entire supercoiled target DNA substrate is consumed. The smear below the linear integration product results from multiple concerted integration events on the same target (Figure 2D), which is possible because the viral DNA ends are in large excess.

### Sequence analysis of the concerted integration products

**Figure 3:**
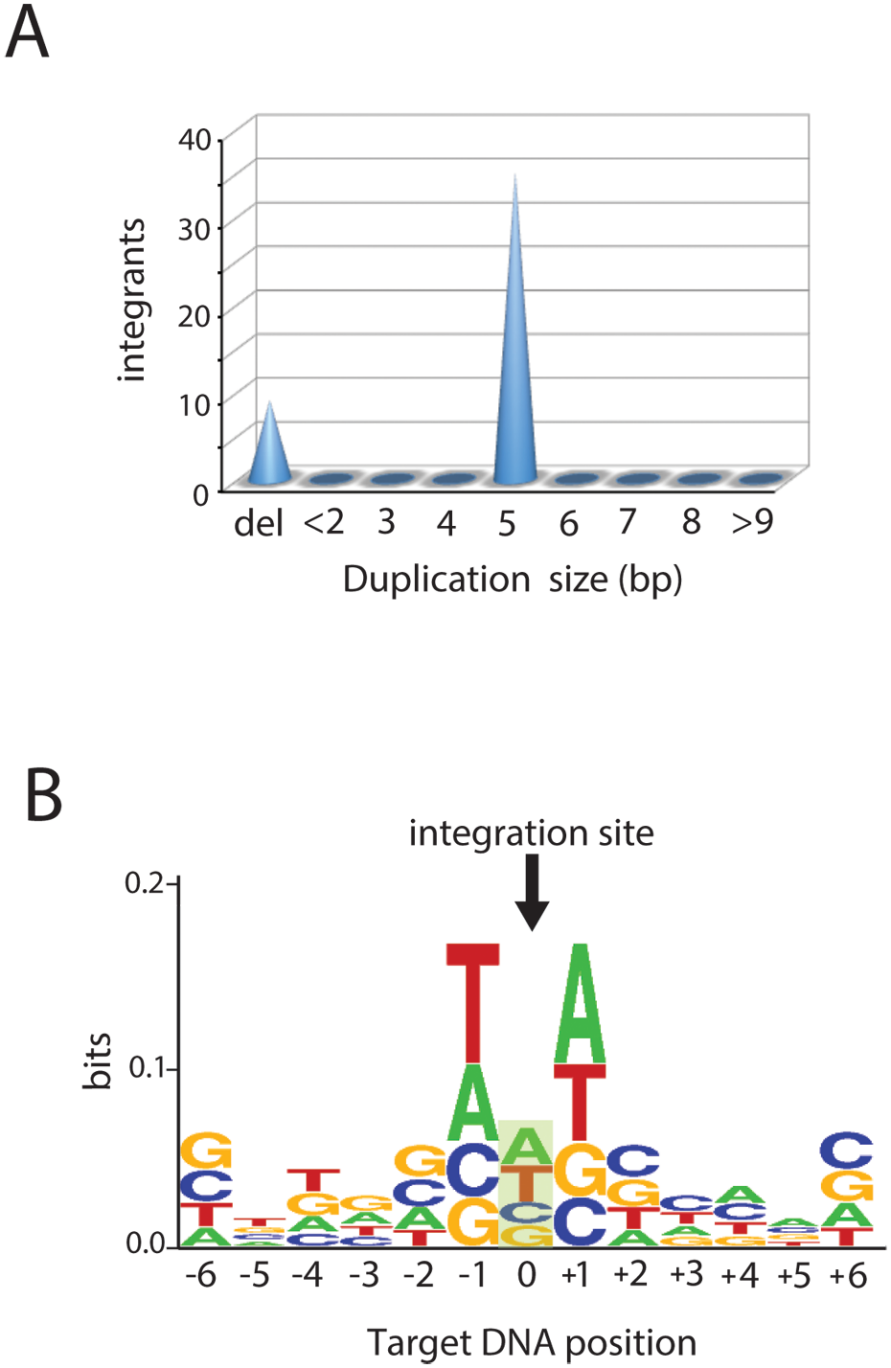
Sequence analysis of concerted integration products. A, Cone presentation of target duplication size distribution or deletions in concerted integrants. 36 clones contained a 5 bp duplication. 10 clones contained a short terminal deletion, likely resulting from contaminating DNA from the smear below the concerted integration product. B, Weblogo representing nucleotide base frequencies at the junction of concerted integration products (n = 72). The arrow indicates the middle position of the 5 bp target site duplication. Alignment of integration site revealed a weak consensus target sequence (GTA/TAC). The overall height of the stack indicates the sequence conservation at that position, while the height of symbols within the stack indicates the relative frequency of each nucleotide at that position. The figure was created by WebLogo.

To test the fidelity of Sso7d-IN mediated strand transfer under optimized *in vitro* conditions the linear DNA corresponding to concerted integration products was isolated from an agarose gel, cloned and sequenced. The majority of integration products contained a 5 bp target site duplication (n = 36), which is the hallmark of correct HIV DNA integration. The other 10 clones did not exhibit target-site duplications and instead had very short deletions of target DNA sequence (Figure 3A). We believe these products are explained by a second concerted integration event occurring very close to the first integration site. In other words, they are representative of the contaminating smear from just below the linear product DNA excised from the agarose gel shown in Figure 2C. Alignment of all the U5-target DNA junction sequences recovered in our experiments (n = 72) revealed a weak bias in nucleotide content that is consistent with the observed *in vivo* results [21] (Figure 3B).

### Sso7d-IN forms stable intasomes with oligonucleotide DNA

**Figure 4:**
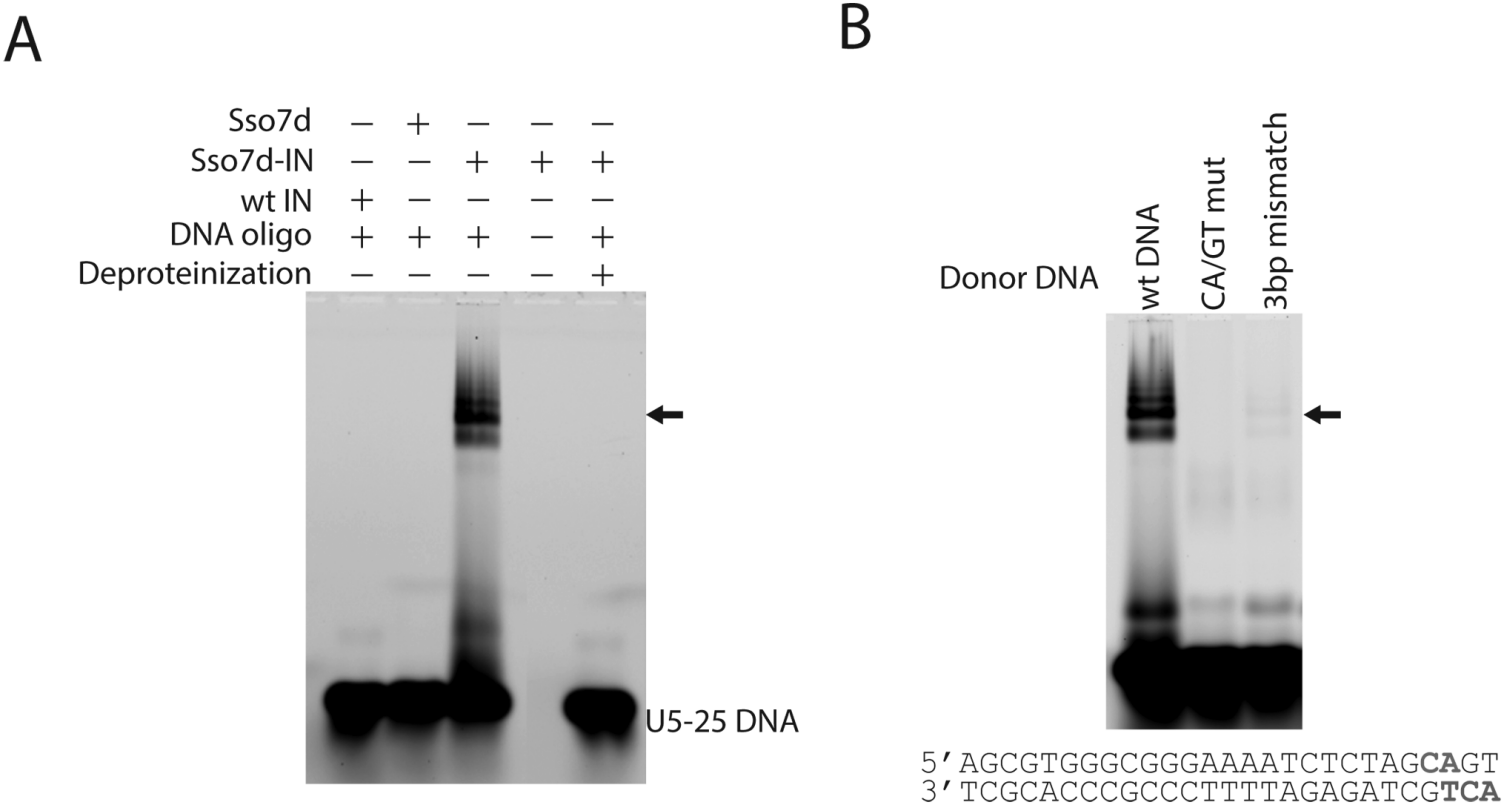
EMSA of intasomes assembled with Sso7d-IN (Gly-11) and a 25 bp DNA substrate (FAM labeled U5-25). To prevent non-specific DNA binding, 10 µg/ml of heparin was added to the reaction mixture after intasome assembly as well as into 3% agarose gels. A, Intasomes assemble with Sso7d-IN (lane 3), but not with wild-type HIV-1 IN (lane 1) or the Sso7d domain alone (lane 2). B, Sso7d-IN specifically assembles intasomes on LTR-U5 sequence (lane 1), but not on “CA/GT mut” (lane 2) or “3 bp mismatch” (lane 3) DNAs. In the “CA/GT mut” DNA, the conserved “CA” dinucleotide is replaced by “GT” (highlighted in the sequence). “3 bp mismatch” was prepared by replacing of “ACT” with “TGA” at the 5′ end of the non-joining strand.

Previous experiments have shown that wild-type IN forms intasomes with 1 kb viral DNA ends and that these intasomes migrate as discrete bands within a native agarose gel [22]. However stable intasomes do not form with DNA shorter than a few hundred base pairs. We therefore tested whether Sso7d-IN forms intasomes with short DNA substrates. Gel-shift analysis showed that Sso7d-IN does indeed assemble intasomes with a 25 bp DNA substrate (Figure 4A). The intasomes were assembled in the presence of Ca^2+^ which supports assembly of intasomes, but not catalysis of DNA strand transfer [23], [24]. Intasome assembly is specific for the viral DNA sequence. Importantly, intasomes were not formed when the conserved CA is mutated to GT or when a 3 bp mismatch is present at the viral DNA terminus (Figure 4B). Intasomes assembled with Sso7d-IN, like PFV intasomes, are stable to challenge with 1 M NaCl (data not shown).

### Efficient strand transfer reactions with purified HIV-1 intasomes

**Figure 5:**
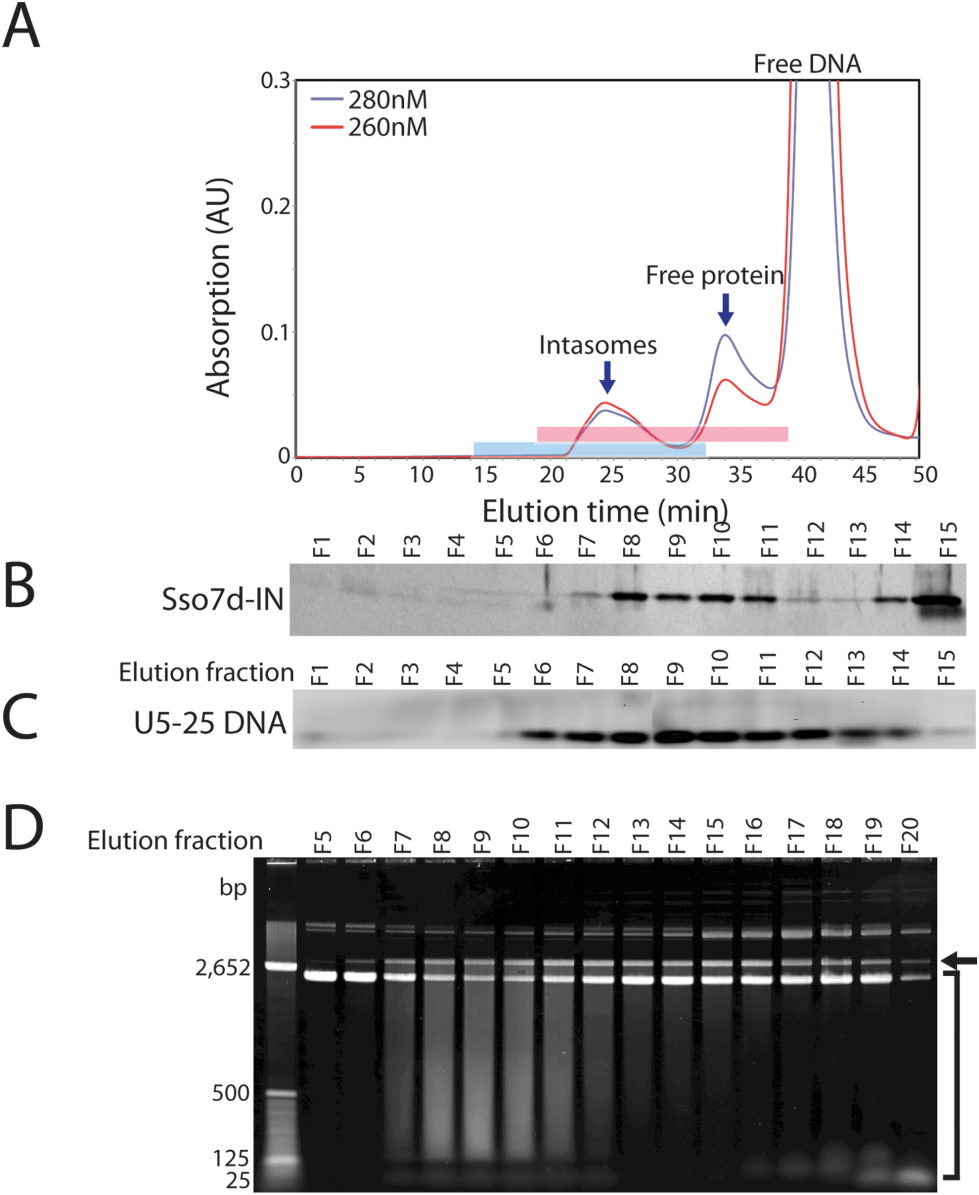
Size-exclusion chromatography of intasomes assembled with Sso7d-IN (Gly-11) and 25 bp DNA (U5-25 DNA). A, Elution profile of intasomes on Superdex 200 2.3/30. The peak labeled free protein as expected elutes in the presence of zinc at a position consistent with a tetramer of Sso7d-IN. The intasome peak elutes at greater than 400 kD relative to protein standards, which is greater than expected for monomeric intasomes. B, Fractions 1 to 15 (F1–F15), corresponding to 13.75 min to 32.5 min elution time (highlighted with blue bar) were analyzed by SDS PAGE (panel B) and 3% agarose gel electrophoresis (panel C), and visualized by silver staining and ethidium bromide staining, respectively. D, Fractions F5 to F20 (highlighted in red), corresponding to 18.75 min to 38.75 min elution time, were tested for strand transfer activity in the presence of Mg^2+^ and supercoiled plasmid DNA. Note that the bulk of strand transfer activity co-elutes with the protein-DNA complex at around 23–28 min elution time (F8–F11). Concerted integration products are indicated by the arrow. The smear resulting from multiple concerted integration events is indicated by the square brackets.

Intasomes were purified from unreacted DNA substrate and free protein by size exclusion chromatography on Superdex 200 (Figure 5A). As expected, the fractions corresponding to the intasome peak contained both DNA substrate and Sso7d-IN protein (Figure 5B and 5C). The purified intasomes carried out highly efficient concerted integration in the presence of Mg^2+^, with minimal half-site integration events (Figure 5D). The smear below the concerted integration product is the result of multiple integrations within the same target DNA molecule.

### Sso7d-IN activity in vivo

**Figure 6:**
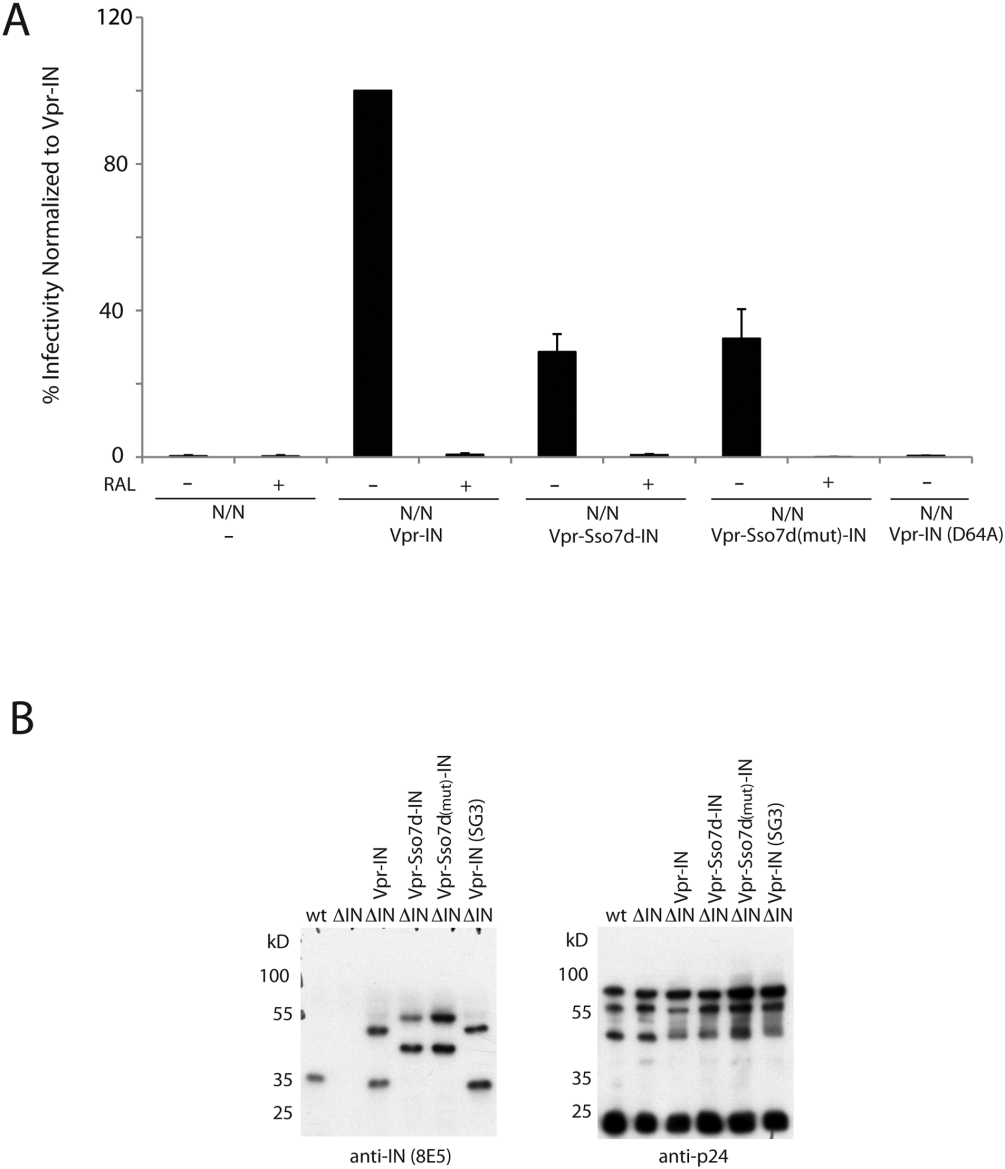
Sso7d-IN is functional in virions. The assay is based on the ability of IN expressed as a Vpr fusion protein to transcomplement N/N virus lacking a functional integrase. A, HIV-1 infectivity normalized to the level obtained with Vpr-IN complementation. The Vpr fusions used for complementation and the infections that were conducted in the presence of RAL are indicated. Sso7d(mut) contains the mutations W24A and R43E which abrogate DNA binding. Graphed are averages with standard deviation for n = 3 (infections with RAL or Vpr-IN-D64A) or n = 6 independent experiments. B, Western blot of IN deletion mutant virus produced with indicated Vpr fusions probed for IN (left panel) and p24 (right panel). All Vpr-IN constructs yielded similar levels of packaged IN protein. The anti-IN antibody 8E5 recognizes the C-terminus (262–271) of IN [18] while the anti-p24 was from Abcam.

Can Sso7d-IN support integration of viral DNA *in vivo*? To answer this question we tested the ability of Vpr-Sso7d-IN fusion protein to transcomplement a non-infectious virus that carries IN active site mutations D64N/D116N (N/N) and a luciferase reporter gene (Figure 6A). As expected (15), the N/N virus alone supported an extremely low level of luciferase activity from unintegrated DNA (∼0.4% of the level of wild-type HIV-Luc infection), which was not further affected by including the potent integrase inhibitor RAL during the infection. The addition of Vpr-IN significantly boosted N/N infectivity to approximately 58% of the wild-type virus. Potent inhibition of this infection by RAL proves that Vpr-IN transcomplementation required HIV-1 DNA integration, which was also evident by the lack of complementation by a Vpr-IN mutant that carried the D64A active site mutation. The function of Vpr-Sso7d-IN was reduced about four-fold relative to wild-type IN. As RAL similarly inhibited this infection, the Sso7d-IN protein is active in the context of HIV-1 infection. Mutations that reduced the binding of Sso7D to DNA had little effect on the efficiency of integration, as is also observed *in vitro*. Western blotting was utilized to visualize IN protein content in virus preparations. To avoid potential misassignement of the viral N/N active site mutant IN, pseudovirus for western blotting was based on an IN deletion mutant construct. Vpr-Sso7d-IN is packaged at comparable levels to that of Vpr-IN (Figure 6B).

## Discussion

Obtaining high-resolution structures of HIV-1 intasomes remains an elusive goal. Such structures are required to understand the detailed mechanism of action of inhibitors and mutations that confer resistance. In the absence of structures of the HIV complexes, models based on the PFV intasome structures are the best available option [11]–[13]. These are likely to be quite reliable in the immediate vicinity of the active site, but because of the degree of sequence divergence, modeling regions further from the active site presents challenges. The two main obstacles to high-resolution structures of HIV-1 intasomes are the propensity of them to aggregate and the requirement for several hundred bp long DNA substrate for efficient assembly. The Sso7d-IN fusion protein described here overcomes the requirement for long DNA through allowing intasomes to assemble with an oligonucleotide DNA substrate as well as improving the solubility of the complex. By a combination of ion exchange and size-exclusion chromatography we have purified HIV intasomes made with Sso7d-IN and concentrated them to about 5 mg/ml in the presence of 0.5 M NaCl (data not shown). However, there is still some heterogeneity as judged by the shape of the peak eluted from size exclusion columns (Figure 5) and atomic force microscopy (data not shown).

How does the Sso7d domain improve the concerted integration activity and biophysical properties of HIV-1 IN? It would appear to not involve DNA binding because these properties are not significantly diminished when the Sso7d domain contains mutations that greatly reduce DNA binding (Figure S1). We favor the hypothesis that Sso7d shields a surface on HIV-1 IN that is responsible for unfavorable interactions that mediate aggregation and non-productive intasome assembly. The hyperactive phenotype likely results from blocking the formation of nonproductive complexes rather than directly “hyperactivating” the enzymatic activity by classical means. This interpretation is consistent with several other observations that are not easily explained. Wild-type HIV-1 IN requires viral DNA several hundred base pairs in length to efficiently assemble intasomes, even though footprinting shows interaction with less than 20 terminal base pairs [22], [25], consistent with the crystal structure of the PFV intasome. Curiously, only one of the two DNA molecules in the intasome needs to be long for efficient assembly [26]. One possible explanation is that the DNA bends back onto the intasome surface masking unfavorable interactions. LEDGF/p75 may also perform a similar function *in vitro*. LEDGF/p75 stimulates concerted integration with oligonucleotide DNA substrate [27] by a mechanism that may be distinct from its *in vivo* role of tethering intasomes to chromatin.

Sso7d-IN is not hyperactive when incorporated into virions and viral DNA integrates with several fold lower efficiency than with wild-type IN. This is consistent with an *in vitro* role of Sso7d in preventing aggregation and non-productive intasome assembly. Since only one viral DNA is made per infecting virion and therefore only a single intasome is assembled, aggregation of intasomes is not an overarching issue and protein modifications that lessen aggregation therefore do not confer an advantage. Although the modified N-terminus hinders IN activity by approximately 4-fold, Sso7d-IN importantly retains significant function in the context of HIV-1 infection. Sso7d-IN is a step forward towards high-resolution structural studies of HIV-1 intasomes, but further advances will likely be necessary before this goal can be attained.

## Supporting Information

Figure S1 **Mutations on the DNA binding surface of Sso7d do not diminish the hyperactive phenotype of Sso7d-IN.** Reactions were carried out with Sso7d-IN or Sso7dmut-IN (W24A/R43E). The DNA substrates were 25 bp of HIV-1 U5 terminal DNA sequence (U5) or the same DNA with a GC rich motif at the 5′ end of the non-transferred strand (see Materials and Methods).
(PDF)

## References

[1] Brown PO (1997) Integration. In: Coffin JM, Hughes SH, Varmus HE, editors. Retroviruses: Cold Spring Harbor Laboratory Press. 161–203.

[2] Hindmarsh P, Ridky T, Reeves R, Andrake M, Skalka AM, et al (1999) HMG protein family members stimulate human immunodeficiency virus type 1 and avian sarcoma virus concerted DNA integration in vitro. J Virol 73: 2994–3003. 10074149 10.1128/jvi.73.4.2994-3003.1999 PMC104059

[3] Li M, Craigie R (2005) Processing the viral DNA ends channels the HIV-1 integration reaction to concerted integration. J Biol Chem 280: 29334–29339. 15958388 10.1074/jbc.M505367200 PMC8742673

[4] Sinha S, Grandgenett DP (2005) Recombinant human immunodeficiency virus type 1 integrase exhibits a capacity for full-site integration in vitro that is comparable to that of purified preintegration complexes from virus-infected cells. J Virol 79: 8208–8216. 15956566 10.1128/JVI.79.13.8208-8216.2005 PMC1143728

[5] Li M, Craigie R (2009) Nucleoprotein complex intermediates in HIV-1 integration. Methods 47: 237–242. 19232539 10.1016/j.ymeth.2009.02.001 PMC3311468

[6] Hare S, Maertens GN, Cherepanov P (2012) 3′-Processing and strand transfer catalysed by retroviral integrase in crystallo. EMBO J 31: 3020–3028. 22580823 10.1038/emboj.2012.118 PMC3395085

[7] Krishnan L, Engelman A (2012) Retroviral Integrase Proteins and HIV-1 DNA Integration. J Biol Chem 287: 40858–40866. 23043109 10.1074/jbc.R112.397760 PMC3510789

[8] Espeseth AS, Felock P, Wolfe A, Witmer M, Grobler J, et al (2000) HIV-1 integrase inhibitors that compete with the target DNA substrate define a unique strand transfer conformation for integrase. Proc Natl Acad Sci USA 97: 11244–11249. 11016953 10.1073/pnas.200139397 PMC17185

[9] Maertens GN, Hare S, Cherepanov P (2010) The mechanism of retroviral integration from X-ray structures of its key intermediates. Nature 468: 326–329. 21068843 10.1038/nature09517 PMC2999894

[10] Hare S, Gupta SS, Valkov E, Engelman A, Cherepanov P (2010) Retroviral intasome assembly and inhibition of DNA strand transfer. Nature 464: 232–236. 20118915 10.1038/nature08784 PMC2837123

[11] Johnson BC, Metifiot M, Ferris A, Pommier Y, Hughes SH (2013) A homology model of HIV-1 integrase and analysis of mutations designed to test the model. J Mol Biol 425: 2133–2146. 23542006 10.1016/j.jmb.2013.03.027 PMC6775779

[12] Krishnan L, Li XA, Naraharisetty HL, Hare S, Cherepanov P, et al (2010) Structure-based modeling of the functional HIV-1 intasome and its inhibition. Proc Natl Acad Sci USA 107: 15910–15915. 20733078 10.1073/pnas.1002346107 PMC2936642

[13] Gupta K, Curtis JE, Krueger S, Hwang Y, Cherepanov P, et al (2012) Solution conformation of prototype foamy virus integrase and its stable synaptic complex with U5 viral DNA. Structure 20: 1918–1928. 23000384 10.1016/j.str.2012.08.023 PMC4034761

[14] Valkov E, Gupta SS, Hare S, Helander A, Roversi P, et al (2009) Functional and structural characterization of the integrase from the prototype foamy virus. Nucleic Acids Res 37: 243–255. 19036793 10.1093/nar/gkn938 PMC2615609

[15] Lu R, Limon A, Devroe E, Silver PA, Cherepanov P, et al (2004) Class II integrase mutants with changes in putative nuclear localization signals are primarily blocked at a postnuclear entry step of human immunodeficiency virus type 1 replication. J Virol 78: 12735–12746. 15542626 10.1128/JVI.78.23.12735-12746.2004 PMC525011

[16] Jurado KA, Wang H, Slaughter A, Feng L, Kessl JJ, et al (2013) Allosteric integrase inhibitor potency is determined through the inhibition of HIV-1 particle maturation. Proc Natl Acad Sci USA 110: 8690–8695. 23610442 10.1073/pnas.1300703110 PMC3666754

[17] Wu XY, Liu HM, Xiao HL, Conway JA, Hunter E, et al (1997) Functional RT and IN incorporated into HIV-1 particles independently of the Gag/Pol precursor protein. EMBO J 16: 5113–5122. 9305652 10.1093/emboj/16.16.5113 PMC1170145

[18] Nilsen BM, Haugan IR, Berg K, Olsen L, Brown PO, et al (1996) Monoclonal antibodies against human immunodeficiency virus type 1 integrase: epitope mapping and differential effects on integrase activities in vitro. J Virol 70: 1580–1587. 8627677 10.1128/jvi.70.3.1580-1587.1996 PMC189980

[19] Gao YG, Su SY, Robinson H, Padmanabhan S, Lim L, et al (1998) The crystal structure of the hyperthermophile chromosomal protein Sso7d bound to DNA. Nat Struct Biol 5: 782–786. 9731772 10.1038/1822

[20] Wang Y, Prosen DE, Mei L, Sullivan JC, Finney M, et al (2004) A novel strategy to engineer DNA polymerases for enhanced processivity and improved performance in vitro. Nucleic Acids Res 32: 1197–1207. 14973201 10.1093/nar/gkh271 PMC373405

[21] Wu XL, Li Y, Crise B, Burgess SM, Munroe DJ (2005) Weak palindromic consensus sequences are a common feature found at the integration target sites of many retroviruses. J Virol 79: 5211–5214. 15795304 10.1128/JVI.79.8.5211-5214.2005 PMC1069554

[22] Li M, Mizuuchi M, Burke TR, Craigie R (2006) Retroviral DNA integration: reaction pathway and critical intermediates. EMBO J 25: 1295–1304. 16482214 10.1038/sj.emboj.7601005 PMC1422164

[23] Ellison V, Brown PO (1994) A stable complex between integrase and viral DNA ends mediates human immunodeficiency virus integration in vitro. Proc Natl Acad Sci USA 91: 7316–7320. 8041787 10.1073/pnas.91.15.7316 PMC44390

[24] Vink C, Lutzke RA, Plasterk RH (1994) Formation of a stable complex between the human immunodeficiency virus integrase protein and viral DNA. Nucleic Acids Res 22: 4103–4110. 7937134 10.1093/nar/22.20.4103 PMC331896

[25] Bera S, Pandey KK, Vora AC, Grandgenett DP (2009) Molecular interactions between HIV-1 integrase and the two viral DNA ends within the synaptic complex that mediates concerted integration. J Mol Biol 389: 183–198. 19362096 10.1016/j.jmb.2009.04.007 PMC2791363

[26] Li M, Ivanov V, Mizuuchi M, Mizuuchi K, Craigie R (2012) DNA requirements for assembly and stability of HIV-1 intasomes. Protein Science 21: 249–257. 22124978 10.1002/pro.2010 PMC3324769

[27] Hare S, Shun MC, Gupta SS, Valkov E, Engelman A, et al (2009) A novel co-crystal structure afords the design of gain-of-function lentiviral integrase mutants in the presence of modified PSIP1/LEDGF/p75. Plos Pathogens 5: e1000259. 19132083 10.1371/journal.ppat.1000259 PMC2606027

