# Supplementary material for: Engineered Hyperactive Integrase for Concerted HIV-1 DNA Integration
Source: PLoS One. 2014 Aug 13;9(8):e105078. doi: 10.1371/journal.pone.0105078 (PMC4132020; doi:10.1371/journal.pone.0105078)
Supplement: Figure S1 — Mutations on the DNA binding surface of Sso7d do not diminish the hyperactive phenotype of Sso7d-IN. Reactions were carried out with Sso7d-IN or Sso7dmut-IN (W24A/R43E). The DNA substrates were 25 bp of HIV-1 U5 terminal DNA sequence (U5) or the same DNA with a GC rich motif at the 5′ end of the non-transferred strand (see Materials and Methods). (PDF) [file pone.0105078.s001.pdf]

Figure S1

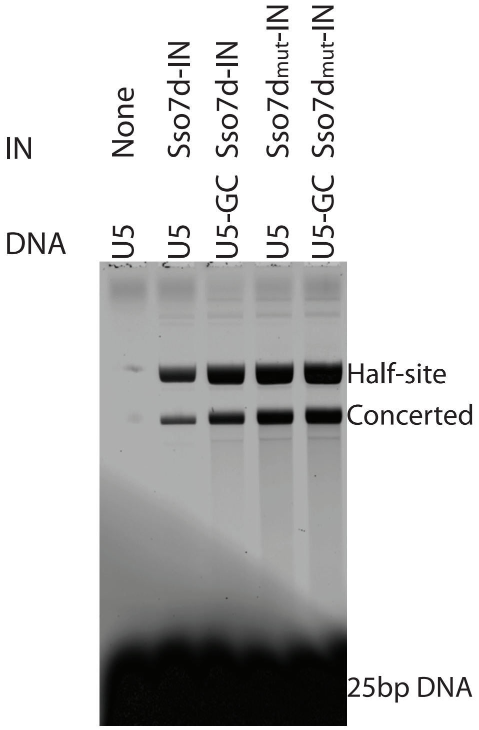
IN
DNA
None
U5
Sso7d-IN
U5
Sso7d-IN
U5-GC
Sso7d$_{mut}$-IN
U5
Sso7d$_{mut}$-IN
U5-GC
Half-site
Concerted
25bp DNA
